# Tell me what to expect: how instructions affect the pain response of patients with chronic myofascial pain with referral

**DOI:** 10.22514/jofph.2024.039

**Published:** 2024-12-12

**Authors:** María García-González, Ignacio Ardizone-García, Laura Jiménez-Ortega

**Affiliations:** ^1^Neuroscience of Emotion Cognition and Nociception Group (NeuroCEN Group), Faculty of Odontology, Complutense University of Madrid, 28040 Madrid, Spain; ^2^Department of Clinical Dentistry, Faculty of Biomedical Sciences, European University of Madrid, 28670 Madrid, Spain; ^3^Psychology and Orofacial Pain Working Group, Spanish Society of Craniomandibular Dysfunction and Orofacial Pain, 28009 Madrid, Spain; ^4^Centre for Human Evolution and Behavior, UCM-ISCIII, 28029 Madrid, Spain

**Keywords:** Pupillometry, Pain expectation, Pain catastrophizing, Orofacial pain, Myofascial pain, Temporomandibular disorders

## Abstract

The aims of the study are to analyze the influence of pain and no pain expectations on the physiological (electromyography (EMG) and pupillometry) and cognitive (Numerical Rating Scale (NRS)) response to pain. Pain expectation and no pain expectation situations were induced by employing instructional videos. The induction of pain was performed by palpating the masseter with an algometer in a sample of 2 groups: 30 healthy participants (control group) and 30 patients (Temporomandibular disorders (TMD) group) with chronic myofascial pain with referral in the masseter muscle (Diagnostic Criteria for Temporomandibular Dissorders (DC/TMD)). Used a mixed design all participants were exposed to pain and no pain conditions in the same session, but the order of the presentation was counterbalanced across participants to control its possible influence. A significantly larger pupillary diameter was observed in the pain expectation relative to the no pain expectation condition in both groups. The TMD group presented larger EMG activity and larger scores in anxiety, somatization, catastrophizing and central sensitization than the control group. In the NRS, the TMD group also showed a significantly higher score than the control group. The TMD group presented similar NRS scores in the expectation condition compared to the no pain expectation condition, while the control group presented higher scores for pain expectation than for no pain expectation. Pain expectation modulated the pain cognitive pain assessment and pupil diameter in controls. The cognitive pain assessment was altered in the TMD group compared to the control group, particularly in the no pain expectation condition, this may be due to a negative reappraisal of pain due to past experiences, as pointed out by the observed level of catastrophizing. Pain expectations did not influence the EMG, significantly higher EMG activity was found in the TMD group compared to the control group regardless of expectation type.

## 1. Introduction

Temporomandibular Disorders (TMD) are defined as a set of musculoskeletal and neuromuscular conditions affecting the temporomandibular joint (TMJ), masticatory muscles and associated structures. They are considered the second most frequent cause of pain in the orofacial region. Among painful TMDs, myofascial pain is the most common TMD disorder, and its etiology is yet to be cleared [1]. Over the last few years, certain psychological characteristics of these patients have been detected as risk and perpetuating factors in myofascial temporomandibular (TM) pain such as anxiety [2, 3, 4], depression and pain catastrophizing [5, 6]. Specifically, regarding pain catastrophizing, it seems that patients with chronic pain present higher levels of catastrophizing and fear of pain [7, 8]. Furthermore, it is considered a possible factor responsible for the chronification of muscular pain in TMD [6, 9].

### 1.1 Pain catastrophizing and pain expectation

Pain catastrophizing refers to the set of exaggerated negative thoughts related to a painful experience or previous to it [10]. It is considered a negative or “maladaptive” pain coping strategy [11]. It influences the increase in suffering [11], the fear of pain [12], as well as the perception of it [8, 13, 14] both by oneself and by others [15]. Additionally, a genetic predisposition might be involved [16].

Pain expectation or fear of pain, which is an important factor of pain catastrophizing, can be induced based on individual previous experiences, verbal instructions about the noxious stimulus and observing other persons suffering pain [17]. Possibly the pain that the individual expects is similar to the perceived one, either due to memory, anticipation or prior conditioning [5, 18, 19]. Similarly, relief expectations would be the basis of the placebo effect [20, 21], as well as distraction [22]. Severeijns *et al*. [23] induced simultaneous pain expectation in healthy participants by verbal instruction using the ice-water hand immersion paradigm. They found no significant differences either in pain perception or in immersion time between the experimental and the control group. However, Fardo *et al*. [24] found that both unexpected (expectation violation) and attended pain-modulated neural gain in the primary and secondary somatosensory cortex (S1 and S2), inferior frontal gyrus, and inferior parietal cortex, applying a dynamic causal model for magnetoencephalography. Neuroimage studies suggest that pain catastrophizing increases activity in the medial prefrontal cortex (anticipation), anterior cingulate cortex and dorsolateral prefrontal cortex (pain modulation), as well as the anterior rostral cingulate cortex, insula and claustrum, closely related to the amygdala (involved in the emotional aspect of pain) [8, 14]. It also can influence modulatory processes of pain inhibition, since it has been observed that the increase in pain catastrophizing produces a decrease in the activity of some regions of the prefrontal cortex [8].

### 1.2 Pain measure: numerical rating scale (NRS) and pupillometry

The Numerical Rating Scale (NRS) has been largely used in the literature to assess pain perception, and there is consensus about its validity [25]. Although the other most used method is the visual analogue scale, both scales are comparable, and numerous studies found a high correlation between the two scales [26].

The pupillometry technique, as a measure of the physiological response to pain, presents a double value. On the one hand, pupil dilatation appears in response to pain, although not exclusively and it is also a recognized measure of the functioning of the Autonomous Nervous System (ANS) [27, 28, 29, 30, 31, 32, 33]. However, recent pieces of evidence show that pupil responses are coordinated by highly interconnected neural circuits beyond the sympathetic system, concerned with attention, alertness, arousal, emotion, executive function and cognition, which involve structures such as the locus coeruleus (norepinephrine system), frontal eye field (FEF) and anterior cingulate cortex (ACC) [34]. Pupil dilation is an evoked reflex and therefore does not involve conscious processing. Consequently, when controlling for confounding factors, such as luminance, it could be an objective pain measurement less influenced by the individual’s cognitive elaboration [35, 36].

### 1.3 Pain perception models

Historically, different models have been described explaining the relationship between chronic muscle pain and muscle function/dysfunction, but at present, it is still not entirely clear. Murray in 2007 proposed a new model called the “Integrated Pain Adaptation Model”, due to the multidimensional nature of pain, he suggests that this motor response depends on the individual experience of pain, and it may therefore be influenced by psychological factors such as catastrophizing and fear or avoidance [37]. In this vein, evidence supports a relationship between psychological factors and the trigeminal motor system, specifically with stress, depression [38] and pain catastrophizing [6, 14, 39, 40]. Furthermore, Bodéré *et al*. [41] believe that the dysfunction observed in patients with chronic painful TMD [42], is not due to nociceptor activation but to Central Nervous System related mechanisms to such a stent that a new approach for treating these disorders could be the modulating neuroplasticity of the emotional and sensorimotor cortex [43].

Present constructivist models of pain perception suggest that, due to the individual’s experience, situation and context, predictive internal models are created and updated considering prediction errors. These internal models would predict (create expectations) and modulate the nociceptive and emotional experience of pain, giving rise to the final perception of pain [44]. Recent studies indicate that factors such as instructions, words, social information and thoughts can activate different mental models of pain and emotions [45], and therefore different pain expectations.

Pain expectation induction and instructions should be considered for patient management and communication, as well as for pain catastrophizing and coping strategies interventions. Therapeutic techniques for both acute and chronic pain management (such as cognitive behavioral therapies [18, 46, 47, 48], mindfulness and relaxation) combine cognitive, attentional and emotional aspects of pain management [46].

Although previous research indicates that pain expectation can be an important factor in the painful experience modulation, to the best of our knowledge, few studies analyze the opposite influence on the placebo effect, that is, the nocebo effect [49] and results are very dissimilar [8, 23].

### 1.4 The present study

The main aim of this study is to analyze the influence of pain and no pain expectation induced by instructions on the physiological (electromyography and pupillometry) and cognitive (NRS) response to experimentally induced pain comparing a group of patients with chronic myofascial pain with referral in the masseter muscle to a control group of participants without myofascial pain, considering the psychological and behavioral characteristics of the participants.

We expect that pain expectancy affects or modulates physiological (pupil size and muscle activity) and cognitive responses (NRS) to experimental pain in the masseter. Differences between patients with chronic myofascial pain with referral in the masseter and controls are also expected.

## 2. BMaterial and methods

### 2.1 Participants

A sample of 60 participants which has been divided into two groups. TMD participants (hereinafter referred to as the “TMD group”) were consecutively recruited from the Orofacial Pain Clinic at the Complutense University of Madrid. Eligibility criteria include myofascial pain with referral in the masseter muscle diagnosis established by DC-TMD [50] criteria and minimum symptom duration longer than 6 months. Controls are Healthy pain-free subjects, students and staff from the Complutense University of Madrid.

The sample size was calculated using Rosner’s [51] methods given the lack of similar studies in the literature. Such calculation was performed for independent samples, assuming a prevalence of muscle TMDs of 16% [1], a power of 0.8 and a value of α = 0.05, resulting in a sample size of n = 29 participants per group. Altogether, our sample size was n = 30 participants per group.

Inclusion criteria for all participants were age between 18 and 60 years, and integrity of the dental arches. Exclusion criteria common to cases and controls were the following: clinical signs of degenerative TMJ pathology; tricyclic antidepressant medication in the 3 months before the study; nonsteroidal anti-inflamatories opioids, corticosteroids and anxiolytics medication in the last 24 hours before the study and masseters and/or temporalis anesthetics infiltration and/or botulinum toxin treatment in the 3 months before the study. Participants under active dental treatment, ophthalmological diseases affecting the biological function or appearance of the eye (*e.g.*, cataracts, nystagmus, amblyopia and macular degeneration) [52], severe psychiatric diseases and pregnant women were also excluded.

The participants’ pain expectation (pain and no pain) was manipulated by two videos of instructions presented before the experimental pain induction. Pain was induced by palpating the participant’s masseter muscle using an algometer with an intensity of 10% above the participant’s pain threshold. The pupillary response and electromyography activity were recorded throughout the process and after palpation, the pain cognitive assessment was quantified using the NRS after palpation. For the data analysis, the experimental time flow was divided into different events. Thus, the pain expectation induction video has been divided into two events (baseline and expectation induction), the moment of palpation will be called the “palpation event”, and immediately after is the “post-palpation event”. When the participant receives the NRS instructions, the event is called the “NRS instructions event”, and finally the period given to the participant to provide the NRS scale score is referred to as the “NRS response event”. These will be described in detail in the experimental procedure section.

### 2.2 Pain expectation induction: pain *vs*. no pain instructions

To maintain the participants’ gaze directed at the monitor and the pupillometer, pain expectation and no pain expectation experimental conditions were induced by instructions videos lasting 17 seconds. The videos were recorded by the same researcher, and they were homogenized in terms of contrast, luminance, colour and volume. The instructions given in the videos for each condition can be seen in Fig. 1.

**Fig. 1.** Transcriptions of the pain expectation video (left) and no pain expectation video (right).

Based on the content, the video was divided into two events for further analysis: the first event, called “baseline video event”, common to both videos, lasts 5 seconds and only the palpation instruction is given in it. The second event is different in both videos, and it introduces the pain expectation or no pain expectation. We have called this second event the “expectation induction event”.

### 2.3 Algometer

To perform a reproducible palpation in the masseter, a 5 kg pressure Baseline® analogue algometer (hydraulic algometer, New York, NY, USA) was used, with a precision of 0.05 kg, palpating with a flat circular surface of 1.52 cm^2^. Palpation is carried out for 5 seconds for both conditions at the same point of the same masseter, left or right in a counterbalanced way across participants, previously marked with an eyeliner. Thus, half of the participants were palpated in the right masseter and the other half in the left masseter, with a single exception in the TMD group: if the diagnosis of myofascial pain in the masseter muscle was unilateral, palpation was performed on that side. However, it was counterbalanced including a participant with pain on the other side. During palpation, the participant is in a resting position. It is performed immediately after the pain expectation and no pain expectation videos. According to the literature, context and environmental situations might affect pain perception more predominantly at intermediate pain intensities, that is at intensities neither too innocuous nor too painful [16, 17, 53]. Therefore, the kilograms used in the palpation for both experimental conditions were calculated according to each participant’s pain threshold, adding 10% weight to the previously assessed threshold (see procedure below). Calibration was performed at the beginning of the experiment, after the EMG electrodes placement. Furthermore, since there weren’t any published studies determining pain threshold to masseter palpation using pupillometry, this intensity was preliminarily tested on seven participants included in the present study. It was finally used in the present study because this intensity produced noticeable changes in pupil diameter avoiding both ground and ceiling effects.

Pain threshold is defined as the level of stimulation at which the individual begins to perceive a sensation as painful [54] or as the pressure necessary to produce a mild pain sensation [55, 56]. The calibration process consisted of exerting pressure with the algometer at the marked point on the masseter. Previously participants were instructed to communicate the point at which he/she “begins to feel mild pain”, warning that he/she should not wait to feel intense pain.

### 2.4 Numerical rating scale (NRS)

The pain cognitive assessment of pain to palpation was evaluated using the NRS to fulfil the ideal conditions required for measuring participants’ pupil diameters, such as red dim light and the participants were previously instructed on the use of the NRS using an 11-second video recorded by the same researcher. This video appears on the monitor right after the palpation. This event is called the “NRS instructions event”. The text played in that video is as follows: “Next when the scale appears on the screen, rate your pain on a scale from 0 to 10 where 0 equals no pain and 10 is the worst pain you can imagine”. To maintain eye fixation in the monitor, participants were informed of their rating right after, while looking at a visual representation of the NRS. This is a numerical scale that goes from 0 to 10; 0 represents the total absence of pain and 10 is the worst pain imaginable.

### 2.5 Pupillometry

Pupil diameter was measured by a Gaze Point® 60 Hz infrared technology pupillometer (Vancouver, BC, Canada). The experiment was carried out in a room without windows illuminated exclusively by a red light (15W, 230V E27 red bulb) at the Complutense University of Madrid School of Dentistry, in the Department of Psychobiology and Behavioral Sciences Methods. Before the experiment, the participants remained in the room for 6 minutes to accommodate the pupil to the room luminance conditions [30]. The pupillometry data were normalized using the *Z*-Score [57]. That is, $Z-score=\frac{(Xi-min)-\bar{x}}{SD}$ where “Xi” is the raw pupil diameter, “min” is the minimum pupil size of the participant, and $\bar{X}$ and SD are the mean and the standard deviation of the participant through the whole procedure. Therefore, the mean value of the pupil diameter corresponds to zero and negative values are below the mean diameter while positive values are above the mean diameter. Only left eye data were analyzed as in the absence of diseases the pupillary reflex is symmetrical [58, 59, 60, 61].

Pupillometric records were performed in the following events: (a) Smallest pupil diameter assessment: Before starting the experiment, the pupil was dazzled with a Riester ri-pen® diagnostic flashlight (Pueblo Rincón, San Juan, PR, USA) for the subsequent normalization of the data, thus obtaining the smallest pupil diameter of each participant. (b) During expectation induction (at baseline video event and expectation induction event) (17 seconds). (c) Palpation event (5 seconds). (d) Post-palpation event, (25 seconds after palpation). (e) NRS Instructions event (11 seconds). (f) pain cognitive assessment (NRS response event) (30 seconds). (g) Rest period (5 minutes) between pain expectation and no pain expectation experimental conditions.

### 2.6 Electromyography (EMG)

For the muscle activity recording we used an electromyograph I-330-C2+ (Minneapolis, MN, USA) from J+J Engineering. The characteristics of the EMG are: amplifiers: Input impedance: 10 Gohm; Notch filter: 50/60 Hz; Maximum bandpass: 1 to 400 Hz; Input channels: 12 or 6 Preamplifier channels: 4 (C2+ 12-Ch); 2 (C2+ 6-Ch); Isolations, Optical: 4000 VAC; Amplifier Failure Protection: 50 μA maximum; Static Discharge Protection: ±15,000 V; Test Electrode Impedance: 250 Ohms at 2 Megoms; EMG Step Frequency: 100 Hz to 400 Hz, 10 Hz to 400 Hz; Input Signal Range: ±500 μV or 2000 μV; R-Wave Filter & Detector: Individual Pulse Update; Interbeat interval (IBI) or heart rate (HR) Output: 40 to 200 beats/minute. Bipolar self-adhesive disposable Foam, Hydrogel disposable surface electrodes with an Ag/AgCl sensor of 10 mm diameter were used and placed on the masseter according to the Surface Electromyography for the Non-Invasive Assessment of Muscles (SENIAM) protocol [62]. Before electrode placement, the skin was cleaned to remove any excess oil and dead cells with alcohol and Nuprep® cream to reduce impedance and Ten20 conductive® cream was applied to the electrodes. The EMG data are expressed in μV and have been normalized by percentage concerning the maximum voluntary contraction of each participant [43, 63, 64, 65, 66, 67]. The EMG recordings were as follows:

(a) at the beginning of the study: resting position and maximum voluntary contraction at maximum intercuspidation (MIC): participants were asked to perform the MIC three times, for 3 seconds for each effort for subsequent normalization of the data. (b) During expectation induction (at baseline video event and expectation induction event) (17 seconds). (c) Palpation event (5 seconds). (d) Post-palpation event, (25 seconds after palpation). (e) NRS Instructions event (11 seconds). (f) pain cognitive assessment (NRS response event) (30 seconds). (g) Rest period (5 minutes) between pain expectation and no pain expectation experimental conditions.

### 2.7 Psychological questionnaires

At the end of the experiment, participants filled the Brief Symptom Inventory 18 (BSI-18) (assessing anxiety, somatization, depression and a general index of symptom severity (GI)), the Pain Catastrophizing Scale (PCS) and Central Sensitization Inventory (CSI) (Part A assesses 25 health-related symptoms common to Central sensitivity syndrome. Part B, which is not scored, asks if one has previously been diagnosed with one or more specific disorders, including seven separate Central sensitivity syndrome) in the validated Spanish versions [68, 69, 70].

### 2.8 Experimental procedure

Participants from both the control and TMD groups were divided into two subgroups, labelled Group A and Group B, based on their entry into the study. This division was conducted in a counterbalanced manner to control the order of condition presentations. Consequently, Group A viewed the pain expectation video followed by the no pain expectation video within the same session, while Group B underwent the reverse order (as depicted in Fig. 2). This ensured that all participants encountered both pain and no pain conditions in a counterbalanced manner during the same session, reducing potential order-related biases.

**Fig. 2.** Experimental procedure for group A (up) and group B. Sec: Seconds; NRS: Numerical Rating Scale.

Once the informed consent has been signed by the participant, the experimental procedure is as follows: (1) Initial preparation: the participant is sitting in front of the pupillometer and the monitor. The electrode is placed in the masseter muscle. The masseter palpation point is marked with an eyeliner in the area that remains free of the electrodes. (2) Pain threshold calculation: it is calculated by palpating the masseter at the marked point with the algometer using increased intensities until the participant reported a feeling of pain. (3) Pupil accommodation period (6 minutes) and red-light illumination for the rest of the experiment. (4) Minimum pupil diameter recording after pupil dazzling and the MIC (3 minutes). (5) Baseline video (5 seconds). (6) Pain expectation induction: group A starts with the pain expectation video, while Group B starts with the no pain expectation induction (17 seconds). (7) Masseter palpation (5 seconds; Intensity = threshold + 10%). (8) Post-palpation period (25 seconds): the word “Palpation” (white letters on a black background) is in the center of the monitor during palpation and post-palpation periods. (9) NRS instruction video presentation (11 seconds). (10) NRS presentation and participant response (30 seconds): The scale is presented in the center of the monitor. (11) Resting period (5 minutes): The word “Rest” is displayed in the center of the monitor (white letters on a black background). (12) The second phase of the experiment: the process is repeated from points 5 to 9, but at point 5 group A watches the “no pain expectation video” while group B watch the “pain expectation video”. (13) End of the experiment: the recording of the pupillometer and electromyography is stopped, and the room light is turned on. (14) Psychological assessment: participants fill out the BSI-18 questionnaire, the PCS and CSI.

### 2.9 Data analysis

Statistical analysis was performed using the SPSS Statistics 25® program (IBM, Armonk, NY, USA) for Windows blindly by an independent statistician. Missing values were calculated by applying the Linear Regression method; the cases and controls were considered independently. Blinks and artefacts were removed by visual inspections. Descriptive statistics were calculated for all variables. A general Mixed design Analysis of variance (ANOVA) including factors Event (6) × Expectation (2) × Group (2) was calculated for pupil dilatation score (*Z*-Score) and Event (5) × Expectation (2) × Group (2) was calculated for masseter electromyographical activity. To further explore data at each event, MIXED ANOVA analyses were calculated including factors Expectation (pain, no pain expectation) × Group (TMD, controls) for both pupil dilation and electromyography activity and the participants’ NRS response. Finally, for the psychological questionnaires and other variables related to the clinical history mean comparison between a Chi-square was calculated for categorical variables and *t*-Student for continuous variables. Values of *p* 0.05 were considered significant differences. Greenhouse-Geisser correction was applied when needed to correct for lack of sphericity.

## 3. Results

### 3.1 Sample characteristics

The TMD group was formed by 2 men and 28 women, and the mean age was 33.10 (±12.3) years. The control group was formed by 3 men and 27 women, and the mean age was 28.27 (±9.8) years. For the means comparison of age and gender, Student’s *t*-tests and Mann-Whitney tests were performed, respectively. In both cases, no significant differences were found (*t*_58_ = 1.6, *p* = 0.1 and *z* = −0.46, *p* = 0.64, for age and gender, respectively). Therefore, it can be concluded that the sample is formed by two homogeneous groups in terms of age and gender. Additionally, a Student’s *t*-test comparing the kilograms used in the palpations between the TMD and control groups did not reveal significant differences either (*t*_58_ = 1.3, *p* = 0.2).

### 3.2 Psychological assessment

As can be seen in Tables 1 and 2, the TMD group showed significantly larger scores on GI (*p* = 0.033), somatization (*p* = 0.009) and anxiety (*p* = 0.008) on the BSI-18 questionnaire; on PCS (*p* = 0.04) and the Part A of CSI (*p*≤ 0.001). Also, on CSI part B, the TMD group significantly showed comorbidity with migraine or tension headaches (*p* = 0.039).

**Table 1.** Mean and standard deviation (SD) of the direct scores obtained in the psychological questionnaires and *t*-Student results for: Brief Symptom Inventory 18 (BSI-18), Pain Catastrophizing Scale (PCS) and Central Sensitization Inventory (CSI).

	Group	N	Mean ± SD	*p*-value
BSI-8 GI*				
	TMD	30	14.57 ± 9.744	0.033
	Control	30	9.17 ± 9.425	
BSI-18 SOMT*				
	TMD	30	5.37 ± 3.837	0.009
	Control	30	2.70 ± 3.771	
BSI-18 DEPR				
	TMD	30	3.40 ± 4.300	0.825
	Control	30	3.17 ± 3.779	
BSI-18 ANX*				
	TMD	30	5.80 ± 3.863	0.008
	Control	30	3.30 ± 3.087	
PCS*				
	TMD	30	15.87 ± 11.482	0.040
	Control	30	10.73 ± 6.705	
CSI Part A*				
	TMD	30	39.00 ± 10.402	<0.001
	Control	30	21.57 ± 7.736	

Homogeneity of variance is assumed in all cases. *Denotes statistically significant difference between TMD group and control group. TMD: Temporomandibular disorders. GI: General Severity Index; SOMT: Somatization; DEPR: Depression; ANX: Anxiety; CSI: Central Sensitization Inventory.

**Table 2.** List of diseases reflected in Part B of the central sensitization inventory.

	Group	Frequencies (%)	*p*-value
Migraine or tension headaches*			
	TMD	12 (40.0%)	0.039
	Control	4 (13.3%)	
Irritable bowel syndrome			
	TMD	1 (3.3%)	1.000
	Control	2 (6.7%)	
Multiple chemical sensitivities			
	TMD	1 (3.3%)	1.000
	Control	0 (0.0%)	
Anxiety or panic attacks			
	TMD	7 (23.3%)	0.506
	Control	4 (13.3%)	
Neck injury (including whiplash)			
	TMD	3 (10.0%)	0.612
	Control	1 (3.3%)	
Depression			
	TMD	4 (13.3%)	0.112
	Control	0 (0.0%)	
Fibromyalgia			
	TMD	2 (6.7%)	0.492
	Control	0 (0.0%)	
Restless leg syndrome			
	TMD	0 (0.0%)	-
	Control	0 (0.0%)	
Chronic fatigue syndrome			
	TMD	0 (0.0%)	-
	Control	0 (0.0%)	

Frequencies and Chi-square test and the corresponding correction with Fisher’s exact test and significant bilateral differences for each disease are offered. *Denotes statistically significant difference between Temporomandibular disorders (TMD) group and control group.

### 3.3 Electromyography

The general mixed ANOVA revealed significant differences in the factors Event (*F*(4, 232) = 2.8; *p* = 0.029; η*_p_*^2^ = 0.045; θ = 0.754) and between groups (*F*(1, 58) = 4.8; *p* = 0.031; η*_p_*^2^ = 0.077; θ = 0.583). Further detailed analyses at each event showed that EMG activity was significantly higher activity in the TMD group than in the control group during the events: baseline video event (*F*(1, 58) = 5.9; *p* = 0.018; η*_p_*^2^ = 0.092; θ = 0.665), expectation induction event (*F*(1, 58) = 4.5; *p* = 0.038; η*_p_*^2^ = 0.072; θ = 0.553), palpation event (*F*(1, 58) = 6.1; *p* = 0.016; η*_p_*^2^ = 0.096; θ = 0.682) and NRS Instructions event (*F*(1, 58) = 4.8; *p* = 0.033; η*_p_*^2^ = 0.076; θ = 0.572). However, no differences were found for other factors and interactions (all *F*s < 2.16, *p* > 0.13) (See Fig. 3).

**Fig. 3.** EMG mean (standardized by percentage concerning the maximum voluntary contraction of each participant in microvolts) throughout the set of events by groups (Temporomandibular disorders (TMD) and controls). The mixed ANOVA revealed that EMG activity was significantly higher activity in the TMD group than the control group during the events: baseline video event (*p* = 0.018), expectation induction event (*p* = 0.038), palpation event (*p* = 0.016) and NRS Instructions event (*p* = 0.033), independently of the type of expectation. The error bars represent standard deviation. *p < 0.05. EMG: electromyography; NRS: Numerical Rating Scale; TMD: Temporomandibular disorders.

### 3.4 Pupillometry

The general mixed ANOVA revealed significant differences in the factors Event (*F*(5, 290) = 1101.4; *p* < 0.0001; η*_p_*^2^ = 0.95; θ = 1), Expectation (*F*(1, 58) = 27.5; *p* < 0.0001; η*_p_*^2^ = 0.32; θ = 0.99), and the interaction Event × Expectation (*F*(5, 290) = 4.1; *p* = 0.005; η*_p_*^2^ = 0.361; θ = 0.88), other factors or interactions did not reach significances (all *F*s < 0.92, *p* > 0.29). Further data analyses revealed that from the expectation induction event onwards, the entire procedure was affected, with significantly greater dilation found under the pain expectation condition relative to no pain expectation condition in the expectation induction event (*F*(1, 58) = 56.9; *p* = 0.0001; η*_p_*^2^ = 0.496; θ = 1), palpation event (*F*(1, 58) = 12.5; *p* = 0.001; η*_p_*^2^ = 0.177; θ = 0.935), post-palpation event (*F*(1, 58) = 19.6; *p* = 0.0001; η*_p_*^2^ = 0.252; θ = 0.992), NRS Instructions event (*F*(1, 58) = 11.9; *p* = 0.001; η*_p_*^2^ = 0.171; θ = 0.925) and NRS response event (*F*(1, 58) = 6.5; *p* = 0.014; η*_p_*^2^ = 0.1; θ = 0.705). However, no differences were found in the group factor or the Expectation × Group interaction (See Table 3 and Fig. 4).

**Table 3.** Mixed ANOVA results for each pupil diameter at each event (using normalized *Z*-score) including Expectative (pain, no pain expectation) and Group (Temporomandibular disorders (TMD), controls) factors and their interaction.

		DF	Quadratic mean	*F*	SIG. (Bilateral)	Effect size η*_p_*^2^	Power θ
Baseline video event							
	Expectative	1.58	0.010	0.069	0.794	0.001	0.058
	Expectative × Group	1.58	0.093	0.609	0.438	0.010	0.120
	Group	1.58	0.003	0.023	0.881	0.000	0.053
Expectation induction event							
	Expectative*	1	2.429	56.973	0.000	0.496	1.000
	Expectative × Group	1	0.087	2.038	0.159	0.034	0.290
	Group	1	0.025	0.937	0.337	0.016	0.158
Palpation event							
	Expectative*	1	0.825	12.492	0.001	0.177	0.935
	Expectative × Group	1	0.002	0.031	0.860	0.001	0.053
	Group	1	0.023	0.524	0.472	0.009	0.110
Post-palpation event							
	Expectative*	1	1.352	19.572	<0.001	0.252	0.992
	Expectative × Group	1	0.001	0.016	0.901	0.000	0.052
	Group	1	0.003	0.126	0.724	0.002	0.064
NRS instructions event							
	Expectative*	1	0.779	11.951	0.001	0.171	0.925
	Expectative × Group	1	0.090	1.378	0.245	0.023	0.211
	Group	1	0.068	1.923	0.171	0.032	0.276
NRS response event							
	Expectative*	1	0.434	6.464	0.014	0.100	0.705
	Expectative × Group	1	0.145	2.157	0.147	0.036	0.303
	Group	1	0.044	1.345	0.251	0.023	0.207

*Denotes statistically significant. DF: degree of freedom; SIG.: Significance; NRS: Numerical Rating Scale.

**Fig. 4.** Pupil diameter (millimeters normalized to *Z*-score) mean and standard deviation throughout the set of events the condition in the pain expectation and no pain expectation condition. The mixed ANOVA revealed significantly greater dilation found under the pain expectation condition relative to no pain expectation condition in the: expectation induction event (*p* = 0.0001), palpation event (*p* = 0.001), post-palpation event (*p* = 0.0001), NRS Instructions event (*p* = 0.001) and NRS response event (*p* = 0.014), independently of the group. The error bars represent standard deviation. NRS: Numerical Rating Scale.

### 3.5 NRS response

The mean NRS score reported by the participants were significantly higher in the TMD group than in the control group (*F*(1, 58) = 10.9; *p* = 0.002; η*_p_*^2^ = 0.158; θ = 0.9). For the Expectation factor, no significant differences were found (*F*(1, 58) = 1.01; *p* = 0.318; η*_p_*^2^ = 0.017; θ = 0.168). However, significances were observed in the interaction of Expectation × Group (*F*(1, 58) = 7.5; *p* = 0.008; η*_p_*^2^ = 0.114; θ = 0.768). *Post-hoc* analyses revealed that the control group presented significant differences between pain and no pain expectations conditions (Δ = 0.65, *p* = 0.01) while no significant differences were observed for the TMD group (Δ = 0.3, *p* = 0.23) (See Table 4 and Fig. 5).

**Table 4.** Mixed ANOVA results of the NRS (numerical rating scale) pain assessment, including factor Expectative (pain, no pain expectation), Group (Temporomandibular disorders (TMD), controls) and Expectative × Group interaction.

	DF	Quadratic Mean	*F*	SIG.	η*_p_*^2^	θ
Expectative	1	0.919	1.016	0.318	0.017	0.168
Expectative × Group*	1	6.769	7.487	0.008	0.114	0.768
Group*	1	49.950	10.879	0.002	0.158	0.900

*Denotes statistically significant. DF: degree of freedom; SIG.: Significance.

**Fig. 5.** Mean scores for the numerical rating scale (NRS) in the pain expectation and no pain expectation conditions after palpation for TMD and control groups. The error bars represent standard deviation. TMD group showed an increased NRS mean score in the no pain expectation condition compared to pain expectation condition, while control group presented higher score in pain expectation compared to the no pain expectation condition (*p* = 0.008). TMD: Temporomandibular disorders.

## 4. Discussion

The main aim of this study is to investigate the influence of pain expectation (pain *vs.* no pain) on electromyography activity, pupil dilation and cognitive assessment (NRS) in response to pain stimulation. A significantly larger pupillary diameter was observed in the pain expectation condition relative to the no pain expectation condition, regardless of whether they were in the TMD group or the control group. For EMG, the TMD group presented significantly higher EMG activity than the control group. In the cognitive assessment of pain, the TMD group showed a significantly higher NRS score than the control group. Additionally, while the control group showed significantly higher NRS scores during the pain expectation compared to no pain expectation conditions, no significant differences were observed within the TMD group.

### 4.1 Electromyography

The data collected on the EMG activity of the masseter muscle area were higher in the TMD group than in the control group throughout the experimental process, but these differences were significant in the baseline video event, expectation induction event, palpation event, and NRS instructions event, with the participant being always in the mandibular resting position. Our data agree with those obtained in the study of Bodéré *et al*. [41] in which they found that at rest, the group of patients with chronic myofascial TM pain showed greater EMG activity than the control group. These differences were not found between the group of patients with TMJ disc pathology and the control group in the aforementioned study.

Numerous studies in the literature have examined the EMG activity response of the masseter muscle to pain, yielding varying results. This variability may arise from factors beyond the direct response of the masseter muscle to pain stimulation. For instance, research suggests that facial expressions of pain [71], which intensify in individuals with chronic TMD, might contribute to these differences [72]. Additionally, studies involving healthy participants observed an increase in facial expression and EMG activity related to higher levels of catastrophizing [73] Henderson *et al*. [14] found that catastrophizing influenced the activity of the primary motor cortex, cerebral cortex and motor trigeminal nucleus during both opening and closing movements after the induction of masseter pain through hypertonic saline injections in healthy individuals. Along the same line, these studies indicate that the elevated EMG activity observed in the TMD group compared to the control group in the present study might be attributed to increased pain facial expressions influenced by catastrophizing. Even though during the video baseline pain is not yet evoked, the mere expectation of palpation may have induced a painful memory in the TMD group, which might have increased the EMG activity. For example, evoking the memory of previous masseter muscle explorations performed in most patients in the clinic while in the control group, the palpation of the masseter muscle does not result in a painful recall. Similarly, during the NRS instruction event, the participant is prompted to recall the pain perceived during palpation, again producing the memory of a painful experience. Furthermore, the post-palpation event was the only one in which significant differences weren’t observed between TMD and control groups. At this event, the participant is in a resting mandibular position without receiving any instructions. Therefore, since the palpation event is over, pain catastrophizing might not affect the facial expression. This interpretation is in line with the study by Stohler *et al*. [74], in which they observed that the recall of a painful experience produced increased EMG activity. Altogether, it’s plausible that the interplay between sensory and motor systems is shaped by the complex interaction of biopsychological factors associated with individual pain experiences, as proposed by Murray in his “Integrated Pain Adaptation Model” [75].

No significant differences were observed between pain expectation and no-pain expectation conditions in EMG activity, to the best of our knowledge few studies investigated pain expectations using EMG and none of them in the facial area. Tétreau *et al*. [76] induced experimental pain in the back and observed that expectations modulated EMG activity on a trunk-extension task. Furthermore, as reported above catastrophizing modulations on mandibular EMG activity were observed for opening and closing movements. In the present experiment, in contrast, participants were told to remain still to maintain pupillometry calibration. This might explain the observed lack of significance.

### 4.2 Pupillometry

The findings suggest that as soon as pain or no pain expectation instructions are given, they influence the entire process. That is, a significantly increased diameter was observed for pain expectation compared to no pain expectation for all events except for the baseline video event, proving the validity of our experimental manipulation. Remarkably, during the palpation event, pupil size was also affected by the expectation induction event, even though the kg used in the palpation (see procedure) were the same in both conditions and all participants were exposed to pain and no pain conditions in the same session since a counterbalanced intrasubject design was applied. This suggests that for both the TMD and control group, the pupil size was not affected by the intensity of the painful palpation itself, but rather by cognitive and emotional variables induced by the instructions. This is in line with the recent International Association for the Study of Pain (IASP) definition of pain [77] and current models of pain perception considering emotional and cognitive influences [47].

Although, some evidence points out that there may be a possible dysregulation of the ANS in patients with painful TMD [78, 79, 80]. In the present study, no significant differences have been found between groups in the pupillary response. However, in addition to the ANS, other cerebral circuits related to attention, executive control, emotions and cognition are involved in pupil response under the same lighting conditions which might explain, at least partially, this lack of differences [44]. To the best of our knowledge, there are only two studies with patients with TMD using pupillometry conducted by Monaco *et al*. [58, 59]. In these two studies significant differences were observed after treatment with Transcutaneous Electrical Nerve Stimulation (TENS) between the chronic myofascial TM pain and the control group [59] and significant differences were also obtained during a forced habitual occlusion task *vs.* rest between arthralgia TMD patients versus the control group [58]. However, these studies, among other methodological differences, did not investigate pain anticipation and their pupil measurement data was not normalized. As a result, their methods are significantly different from ours, making direct comparisons challenging.

### 4.3 NRS

The NRS score was significantly higher in the TMD group than in the control group (*p* = 0.002). Quartana *et al*. [81] observed a decreased pain threshold to mechanical pressure in chronic myofascial TM pain patients compared to the controls, a finding they share with the Prospective Evaluation and Risk Assessment (OPPERA) study [2]. Although peripheral sensitization is a well-known factor influencing pain perception and NRS scores, in the present study its influence might be less significant since the intensity of the painful stimulation was individually calibrated (10% above the participant threshold) to be proportionally similar among participants for both pain and no pain expectation conditions and analyses revealed no differences in kg of palpations between control and TMD group. Altogether, although peripheral sensitization cannot be ruled out completely given its importance for pain perception, the increased NRS score for the TMD group should be better explained by other central factors, such as pain catastrophizing and central sensitization syndromes. In this line, several studies observed in TMD-related pain patients altered brain structure and function involved in sensory, motor, cognitive and emotional brain areas, such as somatosensory cortex, medial and dorsolateral prefrontal cortex, amygdala, insula, anterior cingulate cortex, amygdala and insula among others [82, 83]. Furthermore, as observed in our TMD patient’s central sensitization is characterized by larger levels of anxiety, depression, somatization and catastrophizing [2, 3]. Along the same line, other studies have also observed that participants with higher PCS scores showed higher perceived pain scores [15, 84]. In sum, these observations are compatible with the central sensitization largely described in chronic pain patients.

For the control group, the NRS score was significantly larger under the pain expectation, compared to the no pain expectation condition. However, no significant differences were observed for the TMD group between the no pain expectation condition compared to the pain expectation condition. Data point out, that the instructions promoting no pain expectations were not effective in this group, probably because the previous experiences, catastrophizing and central alterations observed for chronic myofascial TM pain patients [56] are probably stronger than the instructions received. These behaviors could be explained through the attentional model of pain catastrophizing, which postulates that patients who experience high levels of pain catastrophizing have greater attention and negative expectations about the painful stimulus [85]. Furthermore, according to recent constructivist models, pain perception is an actively constructed experience, where prior experience is used to generate expectations and predictions that help to interpret sensory input [44]. Thus, a pain-pre-existent expectation might override the no-pain expectation that we are trying to induce. Finally, a possible clinical application of this finding is that in chronic pain patients who have previous experience with a painful intervention is better to tell them what to expect.

### 4.4 Pupillometry *vs.* NRS

Probably, the most remarkable result of this study is that the control group behaves similarly in both pupil diameter and NRS variables (larger values for pain expectation than for no pain expectation), while the TMD patients presented larger NRS levels regardless of the expectation type than controls, but not differences between pain and no pain expectation. Pupil diameter is a more objective unconscious, almost simultaneous [35, 36] cue of pain which might explain why the TMD and control groups behaved similarly. However, the NRS is a posterior cognitive elaboration and pain re-evaluation, which is a posterior conscious cognitive appraisal of the entire situation. Previous studies have shown that cognitive appraisal and rumination can flexibly modulate self-reported experiences [86] (It should be noticed that ruminations, together with helplessness and magnification are considered pain catastrophizing facets [87]). In this line, an EMG study on the facial muscles found that internal cognitive reappraisal can generate a negative affective response to neutral stimuli [88]. Therefore, as discussed above, the non-pain instructions followed by mild pain palpations might be reinterpreted by TMD patients as more painful than it should be, according to their own pupillometry and control group data. Altogether, our data point out that the most important altered aspect of TMD patients might be the cognitive appraisal of pain. This aspect is rarely taken into consideration in the TMD patient assessment and management, to such an extent that the current DC-TMD axis II does not include any tool to assess and manage cognitive aspects of pain perception.

### 4.5 Limitations

The present study was performed in a controlled environment, the conditions of the room with the lights off and the communication of the instructions to the participants using videos, allow a greater control of the methodology, but the results generalization is lower. It would be interesting to investigate in a clinical rather than laboratory setting, although in that case the pupillometry technique could be used with several limitations since it requires at least dim and constant lighting conditions. However, it would be possible to investigate the influence of pain expectation using additional measures such as skin conductance. Additionally, since pain stimulation was mild and just one intensity was used, it is hard to predict to what degree those results are generalized for other pain intensities. Therefore, future research should investigate the role of different intensities in pain expectations. Nonetheless, the present study offers interesting insights into how instructions affect pain perception which can be useful also in more natural environments since both the dental office and our experimental setup are stressful situations for patients where communication is essential.

## 5. Conclusions

According to recent constructivist models, pain perception is an actively constructed experience, in which emotion, cognition and pain are feedback on each other, where prior experience is used to generate expectations and predictions that help to interpret sensory input. Our data support this view of pain perception pointing out the importance of pain cognitive aspects. A negative cognitive appraisal mediated by catastrophizing and pain cognitive areas alteration due to past experiences might be regulating the TMD participants’ responses, manufacturing an increased pain assessment to no pain instructions followed by a mild masseter pain palpation. Often TMD assessment and treatments do not take into consideration cognitive aspects. Although emotional aspects such as depression and anxiety levels are taken into consideration in the DC-TMD axis II, cognitive aspects of TMD should be also included.

## 6. Key findings

In line with constructivist models of pain perception, our results could show a pre-existent negative pain expectation due to past experiences which might override the induction of no pain expectation by the instructions in the TMD group.

## 7. Clinical implications

Pain expectations together with other cognitive and emotional variables constitute an important element to consider in the management and treatment of both acute and chronic pain. According to our data, special attention should be paid during patient management not only to the way instructions and explanations are given, but also to the cognitive aspect of pain, which nowadays are hardly considered during assessment and TMD treatment and management. Cognitive-behavioral therapies and Mindfulness-based cognitive therapy mindfulness focused on cognitive aspects and promoting positive reappraisal and other strategies reducing catastrophizing and rumination should be considered in TMD treatment.
